# Omics Data Integration: Focusing on Molecular Biomarkers for Cancers and Diseases

**DOI:** 10.3390/biom16060847

**Published:** 2026-06-10

**Authors:** Lucia De Salvo, Cinzia Franchin

**Affiliations:** 1 Department of Pharmaceutical and Pharmacological Sciences, University of Padova, Via F. Marzolo 5, 35131 Padova, Italy; lucia.desalvo@studenti.unipd.it; 2 Mass Spectrometry Facility (MS@DSF), Department of Pharmaceutical and Pharmacological Sciences, University of Padova, Via F. Marzolo 5, 35131 Padova, Italy

## 1. Introduction

In recent years, rapid technological progress in high-throughput sequencing, advanced mass spectrometry, and quantitative imaging has profoundly reshaped biomedical and clinical research. As a consequence, the sheer volume and diversity of available molecular data have grown exponentially, bringing genomics, transcriptomics, proteomics, metabolomics, lipidomics, epigenomics, and metagenomics into routine scientific practice [1]. While each omics layer offers a valuable but partial view of biological systems, it is the integration of these layers that ultimately enables a comprehensive, systems-level interpretation of molecular processes. This transition from single-omic to multi-omic analysis has become a hallmark of modern systems biology and is regarded as a key driver for the discovery of more accurate and functionally informative biomarkers [2].

One of the strengths of the multi-omics approaches is their complementarity of omics technologies. It is therefore noteworthy that outputs from various, yet closely related molecular layers rarely fully correlate. For example, transcriptomic and proteomic data often only partially coincide, as gene expression does not uniformly lead to proportional protein abundance because of post-transcriptional regulation, protein turnover, and degradation mechanisms [3,4]. Therefore, inconsistency of the omics layers is not a technical issue, but rather an indication of the complexity of cellular biology. From a practical point of view, one omics layer is a limited choice and thus conclusions may end up being incomplete or misleading, which could explain why some single-omic biomarkers fail in clinical settings [5]. However, differences across omics layers can themselves provide valuable biological insights, elucidating functionally active pathways, post-transcriptional regulatory mechanisms, and cellular responses to disease or stress. A key reason integrative multi-omics approaches are critical to gain a more comprehensive understanding of disease biology could be attributed to the lack of perfect concordance between genomics, transcriptomics, proteomics, and metabolomics [5,6].

Integrated multi-omics strategies have been particularly transformative in the field of biomarker discovery. Diseases such as cancer, neuroinflammatory disorders, chronic inflammatory conditions or metabolic syndromes are characterized by complex interactions spanning gene regulation, protein networks, metabolic fluxes, and environmental contributions. Relying on a single type of molecular information is often insufficient to fully capture this complexity. Instead, combining transcriptomic signatures with epigenomic marks, proteomic pathways, metabolomic profiles, or imaging-based phenotypes can generate biomarkers that are not only more robust, but also more likely to reflect causal biological mechanisms. Advances in computational biology, machine learning, structural modeling and network science have further expanded the analytical landscape, making it possible to integrate multimodal data in increasingly sophisticated ways [7,8].

This Special Issue of *Biomolecules*, titled “Omics Data Integration: Focusing on Molecular Biomarkers for Cancers and Diseases”, was designed to explore this emerging frontier by gathering original research and review contributions that make explicit use of multi-omics integration or multimodal biomarker approaches. The collection covers a broad range of biological contexts, including prostate cancer, multiple sclerosis, rare metabolic diseases such as alkaptonuria, inflammatory bowel disease-associated cancers, and lymphoid malignancies such as diffuse large B-cell lymphoma (DLBCL). Across these diverse disease landscapes, the authors share a common aim: to leverage integrative analytics to uncover molecular signatures that improve understanding of pathophysiology and support the development of diagnostic, prognostic or therapeutic strategies.

In addition to generating new biological knowledge, this Special Issue also raises several more general methodological and translational challenges of integrating multi-omics. Especially among these studies, the literature demonstrates the importance of standardizing datasets and analysis approaches, and the increasing need for computational techniques able to harmonize heterogeneous data types. At the same time, however, they indicate the continuing difficulty in linking molecular signatures with phenotypic traits and clinically relevant endpoints. Combined, these aspects highlight the increasing focus on the translational applicability of multi-omics research and its potential impact on precision medicine.

Together, the contributions emphasize how integrative omics approaches can provide a more complete and clinically meaningful picture of disease biology, when combined with rigorous methodology and innovative experimental design.

## 2. Overview of Published Papers

### 2.1. Exploring Regulatory Networks in Prostate Cancer Through Transcriptomic and Epigenetic Integration

In their original research article, Schulz and colleagues (paper 1) examine the behavior of prostate cancer cells exposed to simulated microgravity, an unconventional perturbation that modulates cellular architecture, cytoskeletal tension and metabolic activity. By integrating transcriptomic, epigenetic and DNA-methylation data, the authors identify regulatory programs associated with heightened metastatic potential. The study illustrates how environmental perturbations can unmask latent molecular signatures that may not be detectable under standard culture conditions, thereby broadening the scope for biomarker discovery.

This contribution exemplifies how integrating multiple omics layers enables a more nuanced interpretation of molecular adaptation, highlighting pathways that play pivotal roles in prostate cancer progression. Microgravity induced clear morphological changes and altered the expression of genes related to the cytoskeleton, extracellular matrix, cytokines, and major signaling pathways such as PAM, VEGF, and MAPK. Several gravity-sensitive genes with potential relevance as prostate cancer biomarkers were identified, including collagens, integrins, interleukins, and fibronectin.

### 2.2. Combining Molecular Dynamics, Genetic Information and Clinical Data in a Rare Metabolic Disease

Rare diseases, such as alkaptonuria, often suffer from limited patient cohorts and fragmented biological data. This pathology produces a condition in which chronic inflammation driven by homogentisic acid-induced ochronosis is well known but the molecular basis of acute inflammatory flares remains unclear. Trezza et al. (paper 2) address this challenge by employing a clinomics approach, merging genotype data, molecular dynamics simulations, and clinical phenotyping to investigate the role of Serum Amyloid A isoforms in modulating disease severity. Authors demonstrated that patients carrying the SAA1.1 allele exhibit significantly higher levels of acute inflammation, due to the structural instability of the C-terminal region of this protein, which makes it prone to misfolding and fibrils formation. The generated amyloid structures actively stimulate immune pathways, such as Toll-like receptor-mediated cytokine production, creating a self-amplifying feedback loop that increases SAA expression and exacerbates inflammation and secondary amyloidosis.

Their identification of the SAA1.1 allele of Serum Amyloid A as a potential severity biomarker of inflammation in alkaptonuria underscores the value of integrating structural modeling with clinical and molecular observations.

### 2.3. Integrating MRI-Based Phenotyping and Serum Proteomics in Multiple Sclerosis

The work by Jakimovski et al. (paper 3) explores the link between choroid plexus (CP) volume, measured by MRI, and an array of serum biomarkers, including proteomic markers involved in neuroinflammation and neurodegeneration. The study aims to identify blood-based biomarkers associated with CP volumetric changes in multiple sclerosis (MS), examining both cross-sectional relationships and longitudinal predictive effects. A total of 202 people with MS (including relapsing-remitting and progressive forms) underwent brain MRI at baseline and after 5 years, with automated CP segmentation followed by manual refinement. In parallel, serum samples were analyzed using the Olink™ platform to quantify 21 proteins relevant to MS pathophysiology.

The results show that at follow-up, larger CP volume is associated with higher serum neurofilament light chain (NfL) levels and lower osteopontin levels, indicating a link with neuronal injury and inflammatory processes. Furthermore, higher baseline GFAP and lower FLRT2 levels predict CP volume expansion over 5 years, suggesting an early role of glial alterations. Overall, the findings support the idea that CP expansion in MS reflects chronic and compartmentalized inflammation and is closely related to glial activation and neurodegeneration.

Thus, by correlating imaging features with circulating proteins, the authors highlight the potential of multimodal biomarker strategies in multiple sclerosis (MS).

### 2.4. Linking Oncogene-Driven Biology with Metabolomics in DLBCL

In their review, Suman and colleagues (paper 4) explore the potential of metabolomics, with a specific focus on amino acid metabolism, to improve risk stratification, diagnosis, and prognosis in diffuse large B-cell lymphoma (DLBCL), particularly in cases driven by MYC gene alterations. Given the molecular and clinical heterogeneity of DLBCL and the limited number of biomarkers currently applicable in clinical practice, the authors discuss metabolomic profiling as an alternative and complementary approach to detect high-risk patients.

MYC reprograms cellular metabolism in DLBCL and its deregulation profoundly reshapes amino acid uptake and utilization, especially glutamine and essential amino acids. The analysis suggests that glutamine, glutamate, branched-chain amino acids, tryptophan, and tyrosine could serve as accessible metabolic biomarkers for MYC-driven DLBCL.

This conceptual synthesis underscores the importance of viewing oncogenic signaling not only as a transcriptional process, but as a multi-layered perturbation of cellular homeostasis. Metabolomics, in this context, becomes a promising, rapid, and cost-effective strategy for patient stratification and as a foundation for novel therapeutic approaches targeting amino acid transporters and enzymes in aggressive DLBCL.

### 2.5. Proteomics as a Bridge Between Inflammation and Tumorigenesis in IBD-Related Cancer

Chronic inflammatory bowel diseases (IBDs), including Crohn’s disease and Ulcerative Colitis, are increasingly recognized not only for their clinical burden but also for their association with cancer development. In their review, Saccon et al. (paper 5) explore the complex link between chronic intestinal inflammation, current IBD therapies, and cancer risk, with a particular focus on the urgent need for improved, non-invasive screening strategies. The authors highlight how traditional surveillance methods, such as endoscopy and biopsy, remain invasive and poorly tolerated, underscoring the unmet clinical need for reliable biomarkers.

Against this backdrop, the review provides a clear and timely overview of how modern proteomics, especially mass spectrometry-based approaches, are transforming biomarker discovery in IBD-related cancers. Proteomics is particularly well-suited to investigate inflammation-associated tumorigenesis, as it captures dynamic protein networks, cytokine signaling pathways, and post-translational modifications that cannot be inferred from genomic data alone. From colorectal and small bowel cancer to rarer but highly aggressive malignancies such as cholangiocarcinoma, the article critically examines current evidence and emerging protein candidates detectable in stool, blood, saliva, or other biofluids.

By integrating technological advances with clinical perspectives, this review convincingly positions proteomics as a cornerstone of future precision medicine for IBD patients, with the potential to complement or even replace invasive procedures.

## 3. Conclusions

The collection of studies included in this Special Issue highlights that the ultimate power of multi-omics techniques is not in terms of larger datasets but rather in their ability to connect significant biological parameters across the diverse molecular layers of complexity inherent in disease. Instead of the one-off lists of differentially expressed genes, proteins or metabolites, integrated approaches now relate molecular variations with structural changes, immune activation, metabolic rewiring, imaging phenotypes and clinical severity. Most current diseases—e.g., cancer, chronic inflammatory diseases and neurodegenerative diseases as well as metabolic syndromes—are not attributable to a single genetic or molecular alteration. Rather they emerge through complex interrelationships in such biological processes as gene regulation, protein dynamics, metabolic adaptation, immune activation, tissue remodeling and environmental factors. This means that single-omic studies can be more focused on specific or fragmentary information of a disease. The fusion of genomic, transcriptomic, proteomic, metabolomic, structural imaging and clinical data supports the adaptation of the multi-omics-based perspective from purely description to mechanistic interpretation of pathological phenomena. Moreover, the integration of multiple layers of biological information allows for the robustness and explainability of molecular findings while also establishing that these pathways and mechanisms could be linked and hidden from view solely using single analysis. Such integrative and systems-based perspectives are being transformed toward novel biomarker discoveries in biomedicine, as they facilitate deeper insights into disease biology, paving the way for the development of precision medicine methods. The above contributions also demonstrate that integrative omics is gradually beginning to redefine translational research. The molecular signatures are increasingly interpretable in their wider biology and clinical setting leading to possibilities that include earlier detection, advanced patient stratification, prediction of disease advancement, identification of susceptibility to therapy, and development of less invasive monitoring approaches. However, the current Special Issue points towards a number of major impediments that still prevent the clinical application of integrative omics: even though data integration contributes greatly to the depth of narrative and data analytics power, it is still difficult to separate causal biological mechanisms from complex correlations. Likewise, a large number of developed biomarkers and molecular signatures still need to be extensively validated across larger and more diverse patient cohorts before these can be utilized in strong clinical tools. Technical variability, a lack of standardization, variance in sample preparation and analytical platforms, and the intrinsic biological complexity of human diseases constantly influence the reproducibility and comparability of research findings across studies. But the proliferation of large-scale molecular datasets should not lead to simple assumptions that an explosion in data means more accurate biological interpretations. In the absence of rigorous experimental design, effective computational strategy, thorough clinical context-driven interpretation and critical interpretation, integrative analysis can amplify noise and produce misleading associations as well as substantial biological insight. This Special Issue showcases the promising yet challenging nature of integrative omics research. Together, these findings provide a better representation of multidimensional perspectives on the contribution that multidimensional approaches can make to a functional, mechanistic and clinically relevant understanding of disease biology. They also outline the methodological and conceptual issues that are necessary to fully realize the promise of precision medicine.

## 4. Future Perspective

In the future, emerging technologies such as spatial transcriptomics, single-cell multi-omics, next-generation proteomics, and multimodal machine learning will facilitate the accelerating approach to biomarker discovery and improve our understanding of disease biology. The future should make extensive use of a multi-resolution framework for synthesizing molecular, structural, cellular, and clinical information and insights to obtain a broader perspective of disease mechanisms. Integrative omics studies will contribute to the resolution of therapeutic targets for these complex diseases by uncovering the molecular origins of such diseases. The aim is to increase the chances for patients’ recovery and improve treatment development for better personalized therapeutic strategies. Meanwhile, integrative omics is also slowly beginning to redefine translational research itself, promoting strong communication between clinicians, molecular biologists, bioinformaticians, and data scientists. Interdisciplinary collaboration will be key to turning massive biological registries into clinically relevant knowledge and scientific advances. Beyond biomarker discovery, incorporating multi-omics would allow for better early detection of disease, advanced patient stratification, predicting disease progression, identifying potential therapeutic intervention, and minimizing invasive monitoring. However, the full realization of these technologies will also rely not only on technological advancement but also on the development of solid computational workflows, standardized analytical pipelines, accessible data-sharing resources, and well-characterized validation cohorts. Long-term cross-sectional collaboration is, therefore, essential for the successful clinical translation of multi-omics findings to precision medicine.

It is with gratitude that I acknowledge all authors, reviewers, and the editorial staff of *Biomolecules* for their dedication and contribution to this Special Issue. Their collective efforts demonstrate the transformative potential of omics integration for understanding complex diseases and advancing precision medicine.

## Abbreviations

The following abbreviations are used in this manuscript:

DLBCL	Diffuse Large B-Cell Lymphoma
CP	Choroid Plexus
MS	Multiple Sclerosis
IBDs	Inflammatory Bowel Diseases
